# Chemical Ordering in Liquid and Supercooled Ge_2_Sb_2_Te_5_ Phase-Change Materials

**DOI:** 10.3390/ma18163900

**Published:** 2025-08-20

**Authors:** Tae Hoon Lee

**Affiliations:** Department of Advanced Materials Science and Engineering, Kyungpook National University, Daegu 41566, Republic of Korea; thl@knu.ac.kr

**Keywords:** chemical ordering, bond dynamics, liquid Ge_2_Sb_2_Te_5_

## Abstract

The origin of chemical ordering in liquid and supercooled liquid Ge_2_Sb_2_Te_5_ (GST) was investigated using ab initio molecular dynamics (AIMD) simulations. Bond dynamics were analyzed via continuous ($\tau_{\mathrm{con}}$) and intermittent ($\tau_{int}$) lifetimes. The intermittent lifetime ($\tau_{int}$) reveals that chemically ordered Ge-Te and Sb-Te bonds are the most stable, although $\tau_{\mathrm{con}}$ exhibits a stability anomaly. The faster increase of $\tau_{int}$ for these bonds upon cooling explains the overall chemical ordering. A novel ordering mechanism was identified through the analysis of bond separation dynamics. Te-Te ‘wrong’ bonds exhibit a unique dynamic instability, breaking and separating much faster than any other bond type, which actively drives the system towards chemical order. A correlation between lifetime and bond strength, as calculated by the Integrated Crystal Orbital Hamilton Population (ICOHP), supports these dynamic findings. Chemical ordering shows a positive correlation with medium-range structural order, evidenced by the instability of 4-fold rings containing wrong bonds. This study provides a detailed dynamic origin for ordering in liquid GST, highlighting the role of Te-Te bond relaxation.

## 1. Introduction

The structure of the liquid and supercooled phases of chalcogenides has been of great research interest due to its close connection with the structure of its amorphous counterpart [1]. Upon fast cooling, the thermodynamic driving force towards nucleation is overwhelmed by kinetic factors, which results in a kinetically frozen-in liquid-like disordered amorphous structure at glass transition temperature [2]. Understanding the structure and atomic dynamics of the (supercooled) liquid is, hence, the first step towards a unified understanding of glasses and relevant phenomena.

A general observation in the structure of multicomponent chalcogenide liquids is a significant degree of chemical order that is retained even at temperatures significantly above the melting temperature. This propensity for short-range chemical order in liquid and amorphous chalcogenides has been reported through various experimental observations [3,4,5] and confirmed by recent ab initio molecular dynamics (AIMD) simulations [6,7,8]. Such simulations have shown that structural and chemical ordering occur concomitantly during crystallization [7,9], and a correlation between chemical order and the local atomic coordination of tetrahedrally coordinated Ge atoms has also been reported [6,10,11].

While AIMD simulations can provide valuable, accurate information on the chemical ordering trends and early stages of crystallization, they inherently suffer from limitations in system size and accessible simulation time. In particular, these constraints prevent the direct investigation of large-scale phenomena and long-time kinetics. To bridge this gap, alternative computational approaches have been developed. For instance, machine learning interatomic potentials (MLIPs), trained on large DFT databases, have recently emerged as a powerful tool to study the crystallization kinetics of GST with high accuracy over much larger length and time scales [12,13]. However, whether they can accurately capture the dynamics of bond breakage and formation with the same fidelity as AIMD has not yet been comprehensively verified. Furthermore, although coarse-grained models represent another promising direction for studying large-scale relaxation phenomena [14], these models, by design, average over specific atomic interactions and, thus, cannot be used to study the dynamics of individual chemical bonds directly.

Therefore, while acknowledging the power of these large-scale methods, the present study utilizes the high chemical accuracy of AIMD to elucidate the fundamental, microscopic origin of chemical ordering. Although energetic arguments, such as bond strength differences, may help explain the observed chemical ordering, detailed information on the microscopic processes involved has not been thoroughly studied yet. The origin of chemical ordering may be rooted not only in static bond energies, but also in the dynamic behavior of atomic bonds. Specifically, we anticipated that the kinetics of bond formation, breakage, and subsequent atomic relaxation are the key factors that drive the system towards a chemically ordered state.

To investigate this hypothesis, we focus on Ge_2_Sb_2_Te_5_ (GST), one of the prototypical chalcogenide materials for optical/electronic phase-change memory [15,16,17] and neuromorphic applications [18,19,20,21]. GST is well-known for its transition between a disordered amorphous phase and crystalline phases (a metastable face-centered cubic and a stable hexagonal structure) [22,23]. The origin of its unique properties, including ultrafast crystallization speed and large optical/electronic contrast, has been ascribed to its nature of chemical bonding [24,25,26]. Here, we report that chemical ordering in liquid and supercooled liquid GST can be understood by analyzing the unique chemical bonding characteristics and, importantly, the dynamical behavior of each atom-specific bond type. In particular, we will demonstrate the role that Te-Te pair interactions play in establishing short- and medium-range chemical and structural order in liquid and supercooled GST.

## 2. Modeling and Methods

Ab initio molecular dynamics simulations were performed using the VASP simulation package [27]. The liquid, supercooled liquid, and amorphous GST models were generated using a standard melt-quench procedure under periodic boundary conditions. These models, containing either 180 or 300 atoms in the simulation cell, were prepared by melting the system at 2000 K, followed by equilibration (at 1200 K) and quenching (at a rate of −15 K/ps). The Perdew-Burke-Ernzerhof (PBE) generalized gradient approximation [28] was used for the exchange-correlation functional. For all AIMD simulations, the Brillouin zone was sampled only at the gamma point. A plane-wave energy cutoff of 180 eV was used for the AIMD simulations, while a higher cutoff of 300 eV was employed for single-point calculations. For the structural analysis, a chemical bond between two atoms was considered to be formed if the value of the electron localization function (ELF) [29] for that bond was higher than 0.5, unless otherwise specified. A detailed description of the 4-fold rings, including their specific definition and the methods used for their calculation, can be found elsewhere [9].

## 3. Results

### 3.1. Temperature-Dependent Chemical Ordering

**Figure 1:**
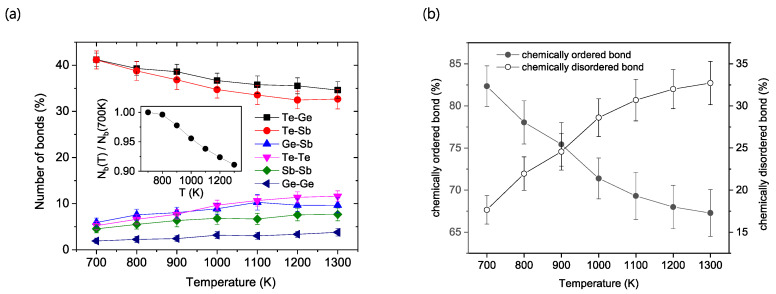
Temperature dependence of chemical-bond populations and chemical ordering in liquid and supercooled liquid GST. (**a**) Percentage of individual chemical bond types as a function of temperature. Upon cooling, the population of chemically ordered bonds (Ge-Te, Sb-Te) increases, while the population of disordered or ‘wrong’ bonds (e.g., Te-Te, Ge-Sb, Sb-Sb) decreases. The inset shows the total number of bonds normalized to the value at 700 K, indicating an increase in the average coordination number with decreasing temperature. (**b**) Total percentage of chemically ordered bonds (Ge-Te and Sb-Te combined) versus chemically disordered bonds (all other types combined) as a function of temperature. The plot clearly reveals a progression towards chemical ordering upon cooling.

The degree of chemical ordering as a function of temperature was first investigated by analyzing the populations of different bond types, as depicted in Figure 1. Figure 1a presents the percentage of each type of bond across the investigated temperature range. As shown in the inset of Figure 1a, a general trend is that the total number of bonds increases as the temperature decreases, indicating an increase in the average atomic coordination number.

When examining individual atom pairs, a clear distinction emerges. The populations of Ge-Te and Sb-Te bonds show a gradual increase upon cooling. These are considered ‘chemically ordered’ bonds, as they are the types observed in the ideal crystalline structure of GST. In contrast, all other bond types—Te-Te, Ge-Sb, Sb-Sb, and Ge-Ge—are classified as ‘chemically disordered’ or ‘wrong’ bonds and exhibit the opposite temperature dependence.

Among the disordered bonds, several interesting observations are made. Te-Te bonds are the most populated type at high temperatures. However, their population diminishes significantly as the temperature decreases, to the point where it becomes similar to the populations of Ge-Sb and Sb-Sb bonds at 700 K. Consequently, the largest reduction in population with decreasing temperature is observed for Te-Te bonds. It is also notable that Ge-Ge bonds consistently remain a minority bond type across all investigated temperatures.

The distinction between ordered and disordered bonds is summarized in Figure 1b, which displays their total percentages as a function of temperature. It is evident that the chemically ordered bonds are the majority species across the entire temperature range investigated, consistently outnumbering the disordered bonds. As the system is cooled from 1300 K to 700 K, the fraction of these ‘correct’ bonds steadily increases, while the fraction of ‘wrong’ bonds correspondingly decreases. This trend clearly illustrates the strong and persistent propensity for chemical order of the system, which is enhanced upon cooling into the supercooled liquid regime.

### 3.2. Bond Dynamics: Lifetimes

**Figure 2:**
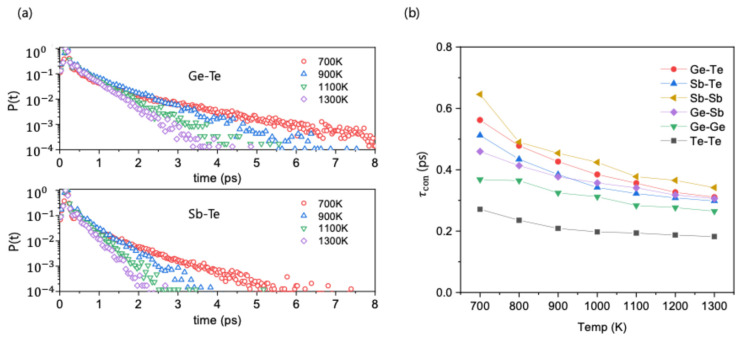
Continuous bond-lifetime ($\tau_{\mathrm{con}}$) analysis. (**a**) The probability density function, P(t), for a bond to persist without breaking, shown for chemically ordered Ge-Te and Sb-Te bonds at different temperatures. For both bond types, the decay of P(t) becomes slower and more non-exponential as the temperature decreases, indicating that bonds survive longer at lower temperatures. (**b**) The continuous lifetime, $\tau_{\mathrm{con}}$, calculated by integrating the P(t) curves, plotted as a function of temperature for all bond types. The analysis reveals an anomaly: the chemically disordered Sb-Sb bond consistently exhibits the highest $\tau_{\mathrm{con}}$. The chemically ordered Ge-Te and Sb-Te bonds have the second and third highest lifetimes, respectively, while the other disordered bonds (Ge-Sb, Ge-Ge, Te-Te) show lower $\tau_{\mathrm{con}}$ values.

To understand the origin of the observed chemical ordering, the dynamic behavior of atomic bonds was investigated using time correlation functions. First, we analyzed the short-time dynamic behavior of bonds by calculating the probability density function P(t) for a bond to persist without any breakage. For the chemically ordered Ge-Te and Sb-Te bonds, the P(t) curves show a non-exponential decay at short times followed by a long-time exponential tail, with the non-exponential character becoming more significant as temperature decreases (Figure 2a). A comparison reveals that Ge-Te bonds decay more slowly than Sb-Te bonds, indicating that, on average, Ge-Te bonds survive longer under thermal fluctuations.

For a quantitative comparison across all bond types, the mean continuous lifetime, $\tau_{\mathrm{con}}$, was calculated using the following relation:

(1) $$\tau_{\mathrm{con}}\;=\int_{0}^{\infty }\mathrm{tP}\left(t\right)\mathrm{dt}.\;$$

This metric, which indicates the fast motion of bonds, represents the average time a bond persists without any breakage. The calculated $\tau_{\mathrm{con}}$ values at 700 K decrease in the order of Sb-Sb > Ge-Te > Sb-Te > Ge-Sb > Ge-Ge > Te-Te (Figure 2b). Although the ordered Ge-Te and Sb-Te bonds are more stable than most disordered bonds, this analysis reveals an anomaly: the chemically disordered Sb-Sb bond exhibits the largest $\tau_{\mathrm{con}}$ value. This high stability of Sb-Sb bonds is inconsistent with their low population seen in Figure 1a, suggesting that other factors beyond the continuous lifetime must play a role in the chemical ordering process.

The continuous lifetime is strongly influenced by frequent bond breakage followed by immediate bond reformation between the same atoms. This limitation makes it less suitable to describe the long-time relaxation behavior of the local structure. To remedy this, we studied the slow bond dynamics using the intermittent lifetime, $\tau_{int}$, from the bond autocorrelation function C(t) [30]:

(2) $$C\left(t\right)=\frac{\langle h\left(0\right)h(t)\rangle }{\langle h(t)\rangle }.\;$$

**Figure 3:**
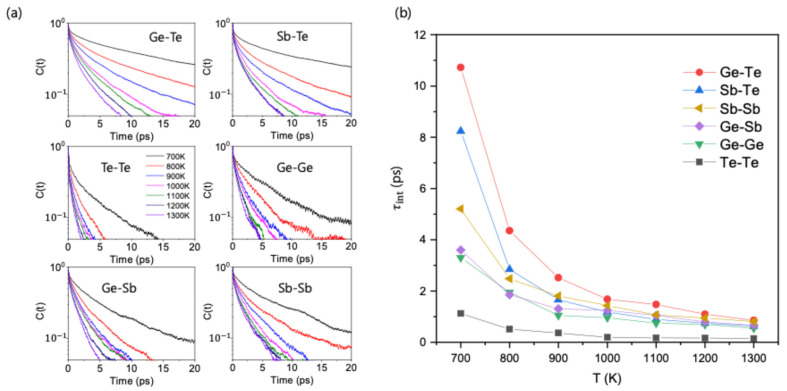
Intermittent bond-lifetime ($\tau_{int}$) analysis as a function of temperature. (**a**) The bond autocorrelation function, C(t), for all six bond types at various temperatures. For all types, the decay of C(t) becomes slower as temperature decreases. Notably, the Te-Te bond curve decays significantly faster than any other type. (**b**) The intermittent lifetime, $\tau_{int}$, plotted as a function of temperature. This lifetime, determined as the time when C(t) decays to 1/e of its initial value, measures long-time structural relaxation by allowing for temporary bond breakage. This analysis resolves the anomaly observed with the continuous lifetime (Figure 2). The chemically ordered Ge-Te and Sb-Te bonds now correctly exhibit the longest lifetimes, followed by the disordered bonds. The plot also shows that the lifetimes of the ordered bonds increase more steeply with decreasing temperature compared to the disordered ones.

Here, h(t) is a dynamical variable, defined as 1 if a specific pair of atoms is bonded at time t, and 0 otherwise. The brackets denote an ensemble average taken over all relevant atomic pairs and multiple time origins. The intermittent bond lifetime is then defined as the time it takes for the autocorrelation function, C(t), to decay to 1/e of its initial value at t=0. Unlike $\tau_{\mathrm{con}}$, the intermittent lifetime (alternatively denoted as the relaxation time, $\tau_{r}$) allows for temporary bond breakage and thus more accurately describes the long-term stability of a chemical bond. The temperature-dependent C(t) curves for each bond type are displayed in Figure 3a.

The calculated $\tau_{int}$ values, depicted in Figure 3b, resolve the anomaly found with $\tau_{\mathrm{con}}$. At 700 K, the order of stability is now Ge-Te > Sb-Te > Sb-Sb > Ge-Sb > Ge-Ge > Te-Te. The chemically ordered Ge-Te and Sb-Te bonds show the largest $\tau_{int}$, followed by the disordered bonds. This revised stability order is now consistent with the observation in Figure 1b, thus successfully explaining the overall increase in chemical order upon cooling. It also explains other details, such as the much smaller population of Ge-Ge bonds compared to Sb-Sb bonds. Another important observation is that Te-Te ‘wrong’ bonds exhibit a noticeably small $\tau_{int}$ compared to any other bond type, a trend similar to the case for $\tau_{\mathrm{con}}$. This consistently high dynamic instability of Te-Te bonds across both short and long timescales suggests that their behavior is distinctly different from all other bond types and may play a unique role in promoting chemical ordering.

### 3.3. Correlation with Bond Strength

**Figure 4:**
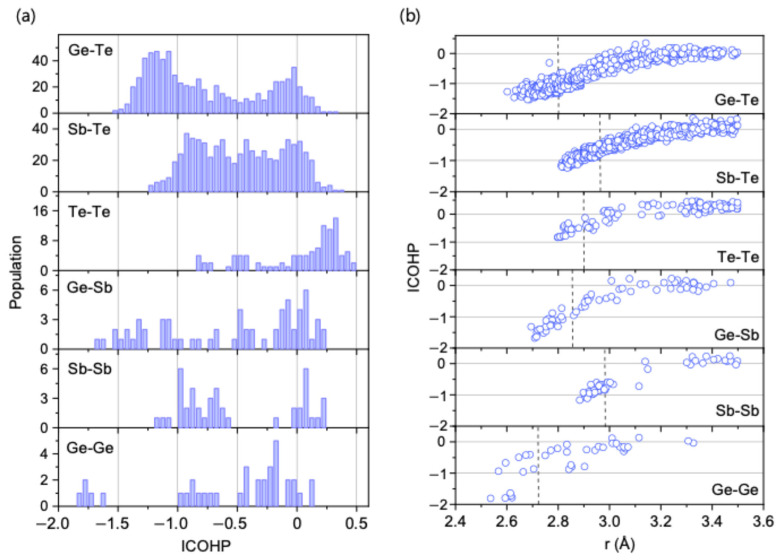
Bond strength analysis using the integrated crystal orbital Hamilton population (ICOHP). (**a**) Population distribution of ICOHP values for each bond type in amorphous GST. Here, a more negative ICOHP value indicates a stronger chemical bond. The histograms show that the chemically ordered Ge-Te (peak at −1.25 eV) and Sb-Te (peak at −0.9 eV) bonds are generally stronger than the chemically disordered Te-Te bonds (peak at −0.75 eV). The exceptions are the islands of very strong bonds observed for Ge-Ge (around −1.75 eV) and Ge-Sb (around −1.5 eV), which correspond to bonds formed by the minority of tetrahedrally coordinated Ge atoms. (**b**) ICOHP values plotted as a function of interatomic distance. The vertical dashed lines indicate the first peak position in the radial distribution function for each bond type, representing the most probable bond lengths.

While the intermittent lifetime ($\tau_{int}$) successfully explains the relative bond populations between chemically ordered and disordered bonds, understanding the underlying physical origins for these different lifetimes requires an analysis of bond strengths. To this end, we calculated the integrated crystal orbital Hamilton population (ICOHP) for each bond type in the amorphous GST models, where a more negative ICOHP value corresponds to a stronger bond (Figure 4).

The analysis reveals that the ICOHP distribution for Ge-Te bonds peaks at a more negative value (−1.25 eV) than that for Sb-Te bonds (−0.9 eV), indicating that Ge-Te forms a stronger bond than Sb-Te. This result corresponds well with the longer lifetimes ($\tau_{\mathrm{con}}$ and $\tau_{int}$) consistently observed for Ge-Te bonds. Furthermore, Te-Te bonds exhibit the least negative ICOHP peak value (at −0.75 eV), which is consistent with their observed lowest lifetimes. Thus, for these primary Ge-Te, Sb-Te, and Te-Te interactions, a clear correlation is established between bond population, dynamic lifetime, and bond strength.

For other disordered bonds, however, the correlation is less direct due to structural heterogeneity. Specifically, some Ge-Ge bonds form an “island” of very strong bonds with ICOHP values near −1.75 eV, and some Ge-Sb bonds appear near −1.5 eV. These strong bonds are characteristic features corresponding to bonds formed by a minority of tetrahedrally coordinated Ge atoms [6,10]. Despite these complexities arising from minority species, the fundamental conclusion from this analysis is that the relatively high bond strengths of the ‘correct’ Ge-Te and Sb-Te bonds, combined with their rapidly increasing intermittent lifetimes upon cooling, provide a physical basis for the overall chemical ordering observed in the system.

### 3.4. Bond Dynamics: Effective Bond Separation

The consistent short lifetimes of Te-Te bonds across both short ($\tau_{\mathrm{con}}$) and long ($\tau_{int}$) timescales suggest that they undergo exceptionally fast structural relaxation. This unique dynamic behavior is potentially a key process associated with the overall chemical ordering. To investigate this mechanism in more detail, we moved beyond lifetime analysis to directly probe the dynamics of bond separation.

**Figure 5:**
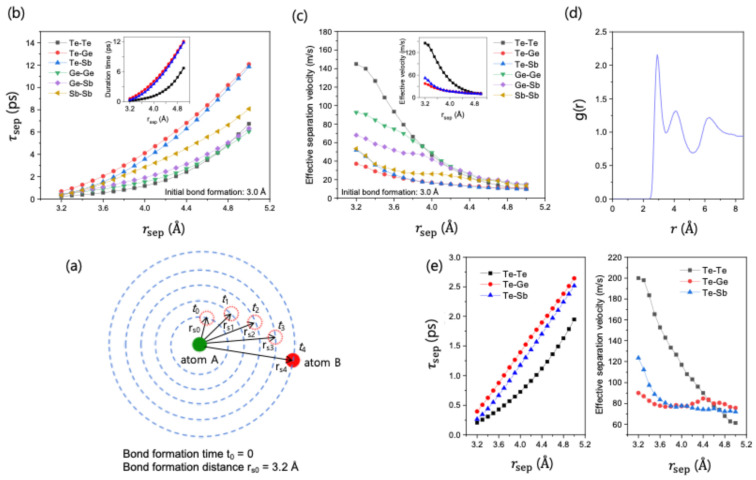
Analysis of dynamic bond separation using separation time ($\tau_{sep}$) and effective separation velocity ($v_{sep}$). (**a**) Schematic illustrating the calculation of $\tau_{sep}$, the time it takes for a newly formed bond pair (defined at t=0) to separate by a distance $r_{sep}$. (**b**) $\tau_{sep}$ as a function of $r_{sep}$ at 700 K. The separation times for Te-Te bonds are the lowest, indicating the fastest separation, while the times for ordered Ge-Te and Sb-Te bonds are the highest. The inset highlights this difference for the key bond types. (**c**) The effective separation velocity, $v_{sep}$ (derived from the slope of the $\tau_{sep}$ curves), as a function of $r_{sep}$ at 700 K. The disordered Te-Te bonds exhibit the highest initial separation velocity, highlighting their strong dynamic instability compared to the chemically ordered bonds. (**d**) The radial distribution function, g(r), for amorphous GST, shown for reference. (**e**) $\tau_{sep}$ (**left**) and $v_{sep}$ (**right**) at a high temperature of 1300 K. The data show that the same qualitative behavior—namely, the uniquely fast separation dynamics of Te-Te bonds—persists even in the liquid phase.

We calculated the effective separation time, $\tau_{sep}$, defined as the time it takes for a pair of atoms, bonded at t=0, to separate by a predefined distance, $r_{sep}$. Figure 5a shows the schematic description of how $\tau_{sep}$ was calculated. The results at 700 K, shown in Figure 5b, reveal that the order of $\tau_{sep}$ values exactly coincides with that of the intermittent lifetimes ($\tau_{int}$), with the ordered Ge-Te bonds having the highest $\tau_{sep}$ and the disordered Te-Te bonds having the lowest. This coincidence indicates that $\tau_{sep}$ effectively captures long-range structural relaxation dynamics. A detailed study on the curves shows that $\tau_{sep}$ for Te-Te pairs increases much less steeply with separation distance than for Ge-Te pairs, indicating that, once a Te-Te bond breaks, the two atoms separate from each other much faster.

To quantify this rate of separation, we defined an effective separation velocity, $v_{sep}$, from the slope of the $\tau_{sep}$ vs. $r_{sep}$ data (Figure 5b). This velocity provides a quantitative measure of how fast a newly broken bond pair separates, with higher velocities indicating a stronger propensity for relaxation away from the disordered, bonded state. As shown in Figure 5c, the order of $v_{sep}$ magnitudes is the inverse of the $\tau_{int}$ order, again confirming that disordered bonds relax faster than ordered ones.

The interesting finding from this analysis is that Te-Te bonds exhibit the highest, initial effective separation velocity of all bond types. This high velocity causes atoms to separate rapidly, then diminishes as their separation approaches the second nearest-neighbor distance (~5 Å), a distance identified from the radial distribution function g(r) (Figure 5d). This process is interpreted as a strong and immediate propensity for structural relaxation whenever two Te atoms are in close proximity. Therefore, this unique dynamic behavior, where ‘wrong’ Te-Te bonds are actively and efficiently removed from the system, can be seen as a natural mechanism that facilitates the chemical ordering process. The same trend is also observed even at 1300 K, well into the liquid phase (Figure 5e), which confirms that the rapid structural relaxation of Te-Te bonds is one of the fundamental mechanisms driving chemical ordering in both the supercooled liquid and liquid phases of GST.

## 4. Discussion

**Figure 6:**
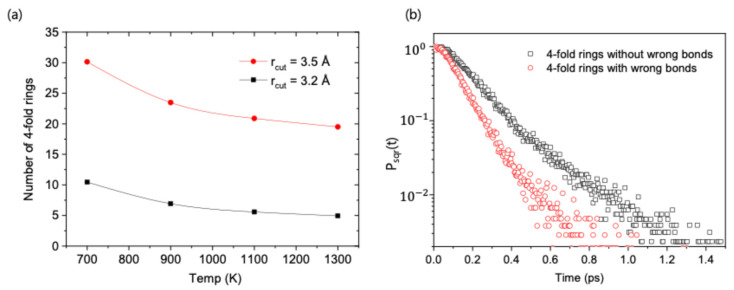
Analysis of medium-range order and its dependence on chemical ordering. (**a**) The number of 4-fold rings as a function of temperature, calculated using two different interatomic cutoff distances (r_cut_) for bond definition. For both cutoff values, the population of 4-fold rings increases upon cooling, indicating a growth in medium-range structural order. (**b**) Probability density for two categories of 4-fold rings, P_sqr_(t): those composed entirely of chemically ordered bonds (‘without wrong bonds’) and those containing at least one disordered bond (‘with wrong bonds’). The slower decay for the chemically ordered rings demonstrates that they are more stable, suggesting a clear relationship between chemical and structural stability.

Chemical ordering at the level of individual bonds, as described in the previous section, also exerts a significant influence on the medium-range structural order of the system. As shown in Figure 6a, the population of 4-fold rings, a primary indicator of medium-range order in GST [7,9], increases as the temperature decreases. This trend occurs in parallel with the chemical ordering detailed previously. Therefore, the stability of these structural motifs appears to be tied to their chemical composition. Figure 6b demonstrates that 4-fold rings containing even a single chemically disordered (‘wrong’) bond are significantly less stable than those composed entirely of ‘correct’ bonds. This indicates a positive correlation between chemical and structural stabilities, suggesting that the formation of stable medium-range structures is favored by adherence to the rules of chemical preference.

Although our simulations are limited to high temperatures (≥700 K), the clear trends in our results, which show increasing chemical and structural ordering upon cooling, strongly suggest that the same underlying mechanisms persist even at lower temperatures. Indeed, this extrapolation is supported by the observations of both significant chemical ordering and a high population of 4-fold rings at 600 K [9].

This interplay between chemical and structural ordering may offer further insight into the well-known ultrafast crystallization of GST [31,32]. Crystallization requires the system not only to arrange atoms into a periodic crystalline lattice, but also to achieve the correct chemical configuration. Our findings point towards a possible dynamic pathway for this process. The rapid dynamic relaxation of Te-Te ‘wrong’ bonds, identified as one of the primary mechanisms for chemical ordering, could be viewed as an important preparatory step for crystallization. By efficiently removing kinetically trapped, chemically unfavorable configurations, the system may be better positioned to form the chemically and structurally stable 4-fold rings, which are known precursors to the crystalline phase [7]. This dynamic error-correction mechanism, driven by the instability of Te-Te bonds, could therefore be a contributing factor in lowering the kinetic barrier for nucleation and enabling the rapid crystallization speed in GST.

The findings presented here support a perspective that extends beyond purely static, energetic arguments to a more dynamic view of ordering in phase-change materials. While bond strength differences are important, the kinetics of bond breakage and relaxation—particularly the rapid removal of unfavorable bonds—appear to be a crucial factor. This dynamic viewpoint may represent a general principle applicable to other multicomponent chalcogenides. A deeper understanding of this atomic-level dynamic behavior could open new avenues for designing next-generation phase-change materials with tailored crystallization speeds and stability for advanced memory and neuromorphic applications.

## 5. Conclusions

In conclusion, we investigated the dynamic origin of chemical ordering in liquid and supercooled liquid Ge_2_Sb_2_Te_5_. An analysis of bond lifetimes revealed that intermittent lifetime ($\tau_{int}$), unlike continuous lifetime ($\tau_{\mathrm{con}}$), correctly identifies the superior stability of chemically ordered Ge-Te and Sb-Te bonds. Their stability increases more rapidly upon cooling, explaining the overall chemical ordering trend. A distinctive mechanism driving ordering was identified through bond separation dynamics: ‘wrong’ Te-Te bonds exhibit a unique dynamic instability. Their rapid relaxation and separation actively drive the system toward a more chemically ordered state. The chemical ordering is positively correlated with medium-range structural stability, as evidenced by the relative instability of 4-fold rings containing ‘wrong’ bonds. This work provides a detailed microscopic picture of ordering in GST, emphasizing that bond dynamics-beyond static properties-are crucial for understanding GST phase-change materials.

## Data Availability

The original contributions presented in this study are included in the article material. Further inquiries can be directed to the corresponding author.
